# Double optimal transport for differential gene regulatory network inference with unpaired samples

**DOI:** 10.1093/bioinformatics/btaf352

**Published:** 2025-08-04

**Authors:** Mengyu Li, Bencong Zhu, Cheng Meng, Xiaodan Fan

**Affiliations:** Institute of Statistics and Big Data, Renmin University of China, Beijing 100872, China; Department of Statistics, The Chinese University of Hong Kong, Hong Kong 999077, China; Center for Applied Statistics, Institute of Statistics and Big Data, Renmin University of China, Beijing 100872, China; Department of Statistics, The Chinese University of Hong Kong, Hong Kong 999077, China

## Abstract

**Motivation:**

Inferring differential gene regulatory networks (GRNs) between different conditions from gene expression profiles remains a significant challenge. Current GRN inference approaches are limited by either scalability in large networks or accuracy in high-dimensional scenarios. Furthermore, most existing methods require paired samples for comparative GRN analyses.

**Results:**

To overcome these challenges, we model gene regulation as a distribution transportation problem and propose an efficient and effective method, called double optimal transport (OT), for reconstructing differential GRNs from the perspective of optimal transport theory, applicable to unpaired samples. Double OT is a novel two-level OT framework. It first aligns unpaired samples by solving a partial OT problem at the sample level, and then infers GRNs from the aligned samples by solving a robust OT problem at the gene level. Comprehensive simulation studies demonstrate the superior efficiency and efficacy of double OT in different scales of networks compared to state-of-the-art methods. We also apply the proposed method to a gastric cancer dataset, identifying the proto-oncogene MET as a central node in the gastric cancer GRN. Its crucial role in early oncogenesis and potential as a therapeutic target further validate our approach and enhance our understanding of the regulatory mechanisms of gastric cancer.

**Availability and implementation:**

A Python library that implements the proposed method is available at https://github.com/Mengyu8042/ot-grn.

## 1 Introduction

Understanding gene regulatory networks (GRNs) is crucial and has broad applications. Reconstruction of GRNs seeks to distill the intricate processes of gene regulation into a simplified network model based on observed data. In this model, nodes represent regulatory and target genes, while edges depict the directional influences exerted by regulators on their targets, grounded in their physical interactions (Delgado and Gómez-Vela 2019). Accurately inferring differential GRNs between different conditions can improve our understanding of gene regulation across different states, such as normal versus tumor tissues, thus illuminating the molecular mechanisms driving diseases and advancing the development of targeted therapeutic interventions (Cangiano *et al.* 2021, Suter *et al.* 2022).

To achieve these goals, a variety of computational approaches have been developed to reconstruct gene regulatory networks from gene expression data. Existing methods generally fall into three categories: correlation-based, model-based, and machine learning-based approaches (Zhao *et al.* 2021, Kim *et al.* 2023). Correlation-based methods use a specific metric such as Pearson’s correlation, Spearman’s rank-based correlation, partial correlation, or conditional mutual information to quantify the association between genes (Friedman *et al.* 2008, Zhao *et al.* 2016, Grimes *et al.* 2019). Although these methods are flexible and computationally efficient, they tend to produce noisy results and lose efficiency when the number of genes significantly exceeds the sample size (Zhao *et al.* 2021). Model-based techniques, on the other hand, employ structured models such as Boolean networks, Bayesian networks, or differential equations, and optimize model parameters to infer relationships (Tsamardinos *et al.* 2006, Haury *et al.* 2012, Yang *et al.* 2021). Such methods offer improved robustness against noise and uncertainty; however, their scalability is limited due to the inherent complexity of the models used (Huynh-Thu and Sanguinetti 2015, Delgado and Gómez-Vela 2019). Lastly, machine learning-based methods reformulate the inference task into classification or regression problems, using algorithms such as random forests, XGBoost, or neural networks to rank the importance of regulatory links (Huynh-Thu *et al.* 2010, Zheng *et al.* 2019, Ma *et al.* 2020). Although powerful, some ML-based approaches are computationally expensive when inferring large-scale networks (e.g. thousands of genes or more), and they may suffer from the curse of dimensionality when only limited sample sizes are available (Delgado and Gómez-Vela 2019, Kim *et al.* 2023). We refer to Badia-I Mompel *et al.* (2023) and Kim *et al.* (2023) for a comprehensive overview.

When the goal is to reconstruct a differential GRN that reflects changes between states or over time (see Section 2 for details), it is natural to extend the approaches mentioned above to comparative study. However, such methods typically require paired samples from different states or time slices, and this requirement is often unmet, resulting unpaired samples. Unpaired samples refer to measurements obtained from distinct cells or patients under different conditions or time slices, where each sample is observed/measured under only one technology, condition, or time slice. For instance, in single-cell RNA sequencing studies, cells are destroyed during sequencing, resulting in unpaired temporal snapshots of cellular states. In addition, when studying changes from normal to tumor states, paired tissue samples from the same individual are often limited. Typically, only tumor samples are accessible for most patients, and normal samples need to be sourced externally. To enhance analytical performance, it is necessary to augment the dataset by matching normal samples from alternative data sources with the tumor samples. An exception that can handle unpaired samples is graphical models (Danaher *et al.* 2014, Tian *et al.* 2016, Tu *et al.* 2021). Instead of directly analyzing changes in gene expression levels, this line of work compares partial correlations defined by precision matrices between different states to infer changes indirectly. More recently, GRN inference methods for single-cell data have also been designed to work with unpaired samples (Demetci *et al.* 2022, Singh *et al.* 2022, Herbach 2023, Bhaskar *et al.* 2024, Zhao *et al.* 2024).

To overcome the limitations of current GRN methods, we develop a novel approach for inferring GRNs based on optimal transport (OT) theory (Villani 2021). Originating from the seminal ideas of Gaspard Monge and later formalized by Leonid Kantorovich, optimal transport aims at moving one distribution of mass to another with minimal effort. Due to its ability to establish correspondences and quantify discrepancies between distributions, OT has been successfully used in various fields, from statistics, economics to biomedical research (Zhang *et al.* 2021, Li *et al.* 2023b, c).

### 1.1 Contributions

Our major contributions are three-fold.

First, by modeling gene regulation as a transportation problem of gene expression distributions, we propose a scalable and effective OT-based differential GRN inference method. Specifically, given comparative gene expression data, we calculate an optimal transport plan to move the distribution of gene expression from one state to another, where the transport mass represents the strength of regulatory relationships. To our knowledge, this is the first work to model gene regulation through the lens of OT theory.

Second, to deal with unpaired data, we introduce a novel two-level OT framework that first applies OT to align unpaired samples at the sample level, followed by OT at the gene level to reconstruct differential GRNs from these aligned samples. Such integration fully leverages the advantages of OT in distribution matching and comparison, and enhances the feasibility of inferring complex biological networks.

Third, we demonstrate the improved accuracy and efficiency of our method through extensive experiments on synthetic data and real-world gastric cancer datasets (Wang *et al.* 2014, Kang *et al.* 2022). Additionally, we identify the proto-oncogene MET as a central node in the gastric cancer GRN, further validating our approach and deepening our understanding of the regulatory mechanisms in gastric cancer.

## 2 Materials and methods

**Figure 1.**
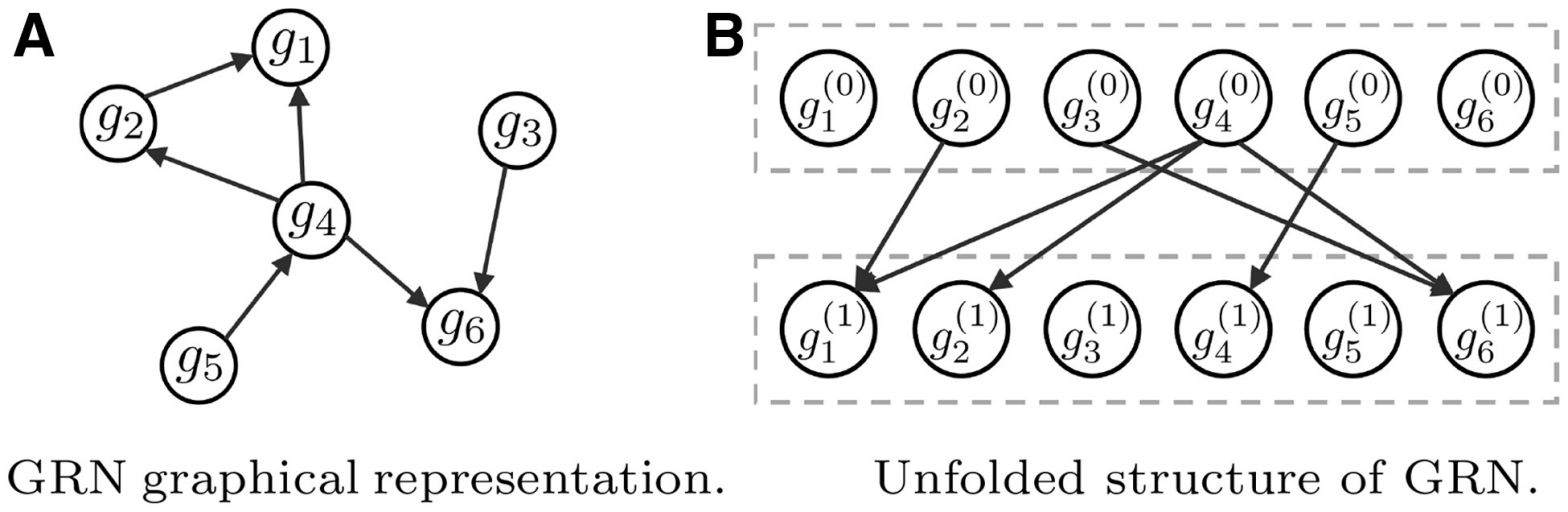
Two equivalent representations of a GRN. In (B), $g_{i}^{\left(t\right)}$ corresponds to the *i*th gene in the *t-*th time slice. For example, t=0 and t=1 represent normal and tumor states, respectively.

We analyze gene expression data from two comparative states (e.g. normal and tumor), represented as $X\in R^{p\times n}$ and $Y\in R^{p\times m}$, respectively. Both datasets contain the same *p* genes but may have unpaired *n* and *m* samples. The target network G=(V,E) is an unsigned directed graph, where *V* represents a set of *p* nodes corresponding to genes $\left\{g_{1},\ldots ,g_{p}\right\}$, and $E\subseteq \{(g_{i},g_{j}):(g_{i},g_{j})\in V^{2}\}$ is a set of directed edges. An edge from node $g_{i}$ to node $g_{j}$ signifies that gene *i* regulates the expression of gene *j* through either activation or inhibition (see Fig. 1A). To depict the dynamic transition from normal to tumor states, we can also reformat the graph in an unfolded structure, as shown in Fig. 1B. This representation explicitly displays the expression relationships between potential regulators and their target genes across different time slices.

Building on established work (Huynh-Thu and Sanguinetti 2015, Zheng *et al.* 2019), we focus on providing a ranking of regulatory links, while deferring the problem of automatically determining a weight threshold for practical network construction to future investigations.

### 2.1 Optimal transport problems

**Figure 2.**
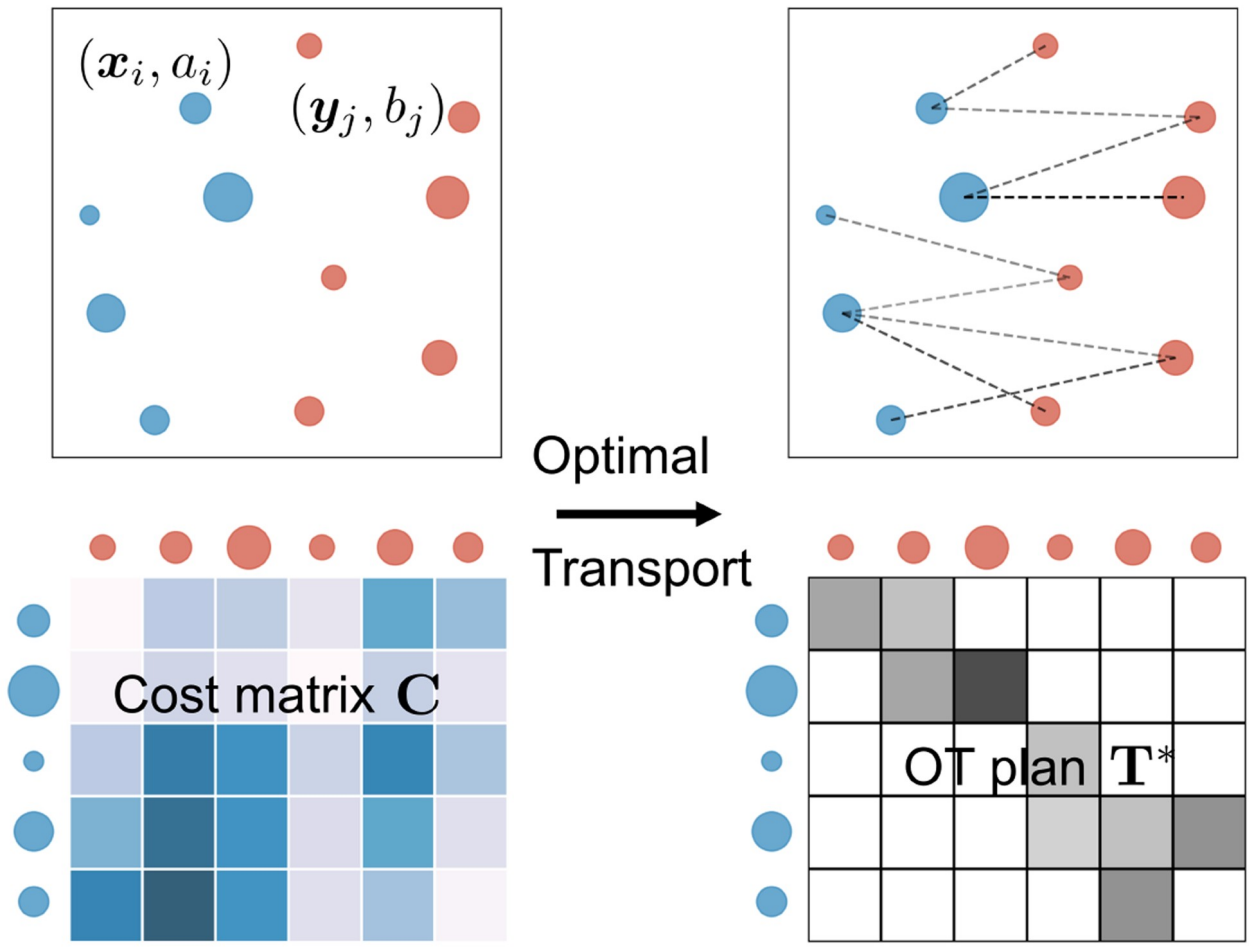
Illustration of optimal transport for distribution matching. (Top row) Blue and red dots represent source and target 2D samples, respectively, with dot size corresponding to mass. (Bottom row) Darker cells indicate higher transportation costs (left) or larger transported mass (right).

Optimal transport has been widely used for distribution comparison and matching (Meng *et al.* 2020, Li M *et al.* 2023a, Li T *et al.* 2024, 2025). Consider two distributions represented by empirical samples ${\left\{x_{i}\right\}}_{i=1}^{n_{1}},{\left\{y_{j}\right\}}_{j=1}^{n_{2}}\subset R^{d}$ with associated mass vectors $a\in R_{+}^{n_{1}}$ and $b\in R_{+}^{n_{2}}$, referred to as source and target distributions, respectively. We can infer their correspondence relationships by solving an optimal transport problem. In particular, the Kantorovich OT formulation is expressed as

(1) $$\begin{array}{c} \min_{T\geq 0}\langle C,T\rangle :=\sum_{i,j}C_{ij}T_{ij}\\ s.t.\;\;T1_{n_{2}}=a,\;\;T^{⊤}1_{n_{1}}=b, \end{array}$$

where $C\in R_{+}^{n_{1}\times n_{2}}$ is a cost matrix derived from the cost function $c:R^{d}\times R^{d}\to R_{+}$, with elements $C_{ij}=c(x_{i},y_{j})$ representing the cost of moving unit mass from $x_{i}$ to $y_{j}$. The matrix $T\in R_{+}^{n_{1}\times n_{2}}$ represents feasible transport plans, where $T_{ij}$ specifies the amount of mass transferred from $x_{i}$ to $y_{j}$. The solution to (1), known as the optimal transport plan, minimizes the total transportation cost. An illustration of the OT framework is provided in Fig. 2.

**Figure 3.**
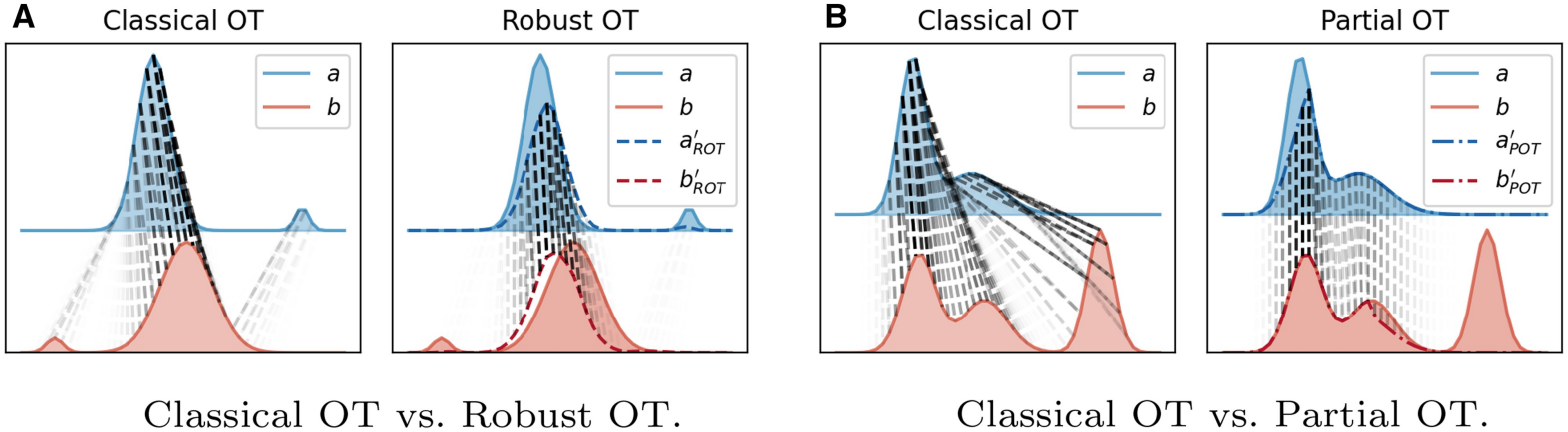
Comparison between classical OT and its variants. a and b represent source and target distributions, respectively. $a_{\mathrm{ROT}}^{\prime }$ and $b_{\mathrm{ROT}}^{\prime }$ in A (or $a_{\mathrm{POT}}^{\prime }$ and $b_{\mathrm{POT}}^{\prime }$ in B) are the marginals of the optimal plan in robust OT (or partial OT) problems.

To address potential outliers and noise in data, robust OT, also called unbalanced OT (Pham *et al.* 2020, Shen *et al.* 2021), relaxes the strict mass conservation constraints in (1) using the Kullback–Leibler (KL) divergence as regularization terms, leading to the formulation:

(2) $$\min_{T\geq 0}\langle C,T\rangle +\varepsilon \text{KL}(T1_{n_{2}}||a)+\varepsilon \text{KL}(T^{⊤}1_{n_{1}}||b),$$

where ε>0 is a marginal relaxation parameter. As ε→+∞, the robust OT problem (2) converges to the classical OT formulation (1), which requires exact mass matching between distributions. As shown in Fig. 3A, robust OT restricts long-range mass transportation compared to classical OT, thus improving robustness in the presence of noisy or outlier data.

Another variant, partial OT, minimizes the transport cost for only a predefined fraction of total mass (Chapel *et al.* 2020). Its mathematical expression is

(3) $$\begin{array}{c} \min_{T\geq 0}\langle C,T\rangle \\ s.t.\;\;T1_{n_{2}}\leq a,\;T^{⊤}1_{n_{1}}\leq b,\;1_{n_{1}}^{⊤}T1_{n_{2}}\leq s, \end{array}$$

where $0\leq s\leq \min(||a|{|}_{1},||b|{|}_{1})$ bounds the total amount of mass to be transported, thereby especially suited for partial or local alignments (see Fig. 3B).

Remark 1 *Although both robust and partial OT allow transporting less than the total mass, they differ in how this relaxation is handled. Partial OT (*3*) imposes a hard transport budget s and can yield sparse one-to-one matches, while robust OT (*2*) uses soft KL penalties and typically yields spread-out transport plans. Therefore, robust OT is better suited for handling noise and outliers (Fig. 3A), whereas partial OT is more appropriate for confidently aligning a subset of samples (Fig. 3B).*

Overall, these optimal transport problems can be unified under a general formulation; see Supplementary Section S1 for details, available as supplementary data at *Bioinformatics* online.

### 2.2 Gene-level OT for GRN inference

In all living cells, resources such as energy, ribosomes, and proteome capacity are finite. The reduced demand for any of these finite resources allows their reallocation to other intracellular processes (Weiße *et al.* 2015). Such “shifting” of resources makes OT at the gene level a natural framework for modeling gene expression changes between two phenotypes (such as normal and disease), which may reveal the gene regulation relationship.

In tissues under normal conditions, certain pathways are stably activated, implying that relative gene expression levels are also stable across different individuals. We represent the normal state by a vector of gene expression proportions, $a={\left(a_{1},\ldots ,a_{p}\right)}^{⊤}$ with $\sum_{i=1}^{p}a_{i}=1$. Similarly for the tumor state, we introduce another state vector $b={\left(b_{1},\ldots ,b_{p}\right)}^{⊤}$ with $\sum_{i=1}^{p}b_{i}=1$. The transition between these two states captures how tumor cells deactivate certain pathways (i.e. genes turned off) and activate alternative pathways (i.e. genes turned on). Genes in these competitive pathways form differential regulatory networks, which offer new insights into the underlying regulatory mechanisms beyond traditional transcription factor (TF)-based interactions.

We first consider the scenario where n=m and the samples in X and Y are paired. Let $x_{\left(i\right)}$ represent the *i*th row of the normal expression matrix X, indicating the expression level of gene *i* in X, with $y_{\left(i\right)}$ similarly defined for the tumor expression matrix Y. Empirically, we use the average expression level of each gene in the normal (or tumor) state as the source (or target) distribution, i.e. $a=X1_{n}/n$ and $b=Y1_{n}/n$.

In GRNs, correlation measures are widely used to infer dependencies between genes. A high absolute correlation indicates a regulatory relationship. Therefore, we define the cost matrix $C\in R^{p\times p}$ with $C_{ij}=c(x_{\left(i\right)},y_{\left(j\right)})=1-|r(x_{\left(i\right)},y_{\left(j\right)})|$, where $r(x_{\left(i\right)},y_{\left(j\right)})$ is the Spearman’s rank-based correlation coefficient between gene *i* in the normal state and gene *j* in the tumor state. This cost ensures that more highly correlated gene pairs have lower transport costs, allowing OT to transport a larger amount of mass, which can be interpreted as stronger regulatory interactions.

Compared to other similarity measures such as Euclidean distance, Pearson’s correlation, and partial correlation, Spearman’s correlation not only captures both linear/nonlinear and positive/negative regulatory relationships, making it suitable for complex biological interactions, but is also computationally efficient. By incorporating this correlation metric into the OT framework, we can effectively infer both the strength and directionality of regulatory interactions. Such correlation-based cost has also been used in OT literature for measuring cell similarities (Huizing *et al.* 2022).

To account for technical noise in data, we solve the robust OT problem (2) at the gene level and obtain the optimal transport plan $T^{*}$, where each element $T_{ij}^{*}$ represents the amount of mass transported from gene *i* to gene *j*, for i,j∈{1,…,p}. A larger $T_{ij}^{*}$ indicates a significant regulatory link from gene *i* to gene *j*, suggesting that this interaction plays an important role in the transition from normal to tumor. Therefore, an edge from gene *i* to gene *j* is established if $T_{ij}^{*}$ exceeds a certain threshold. Genes *i* and *j* connected by such edges are identified as important, potentially influential regulators or key targets.

Remark 2 *The constructed network may contain loops and cycles, because we do not impose restrictions on the structure of the transport plan. Instead, it captures all significant regulatory relationships. Unlike many GRN inference algorithms that generate directed acyclic graphs, our method aligns with the fact that biological networks often contain feedback loops and cyclic interactions (* Hasty *et al.* 2001, Alon 2007*).*

**Figure 4.**
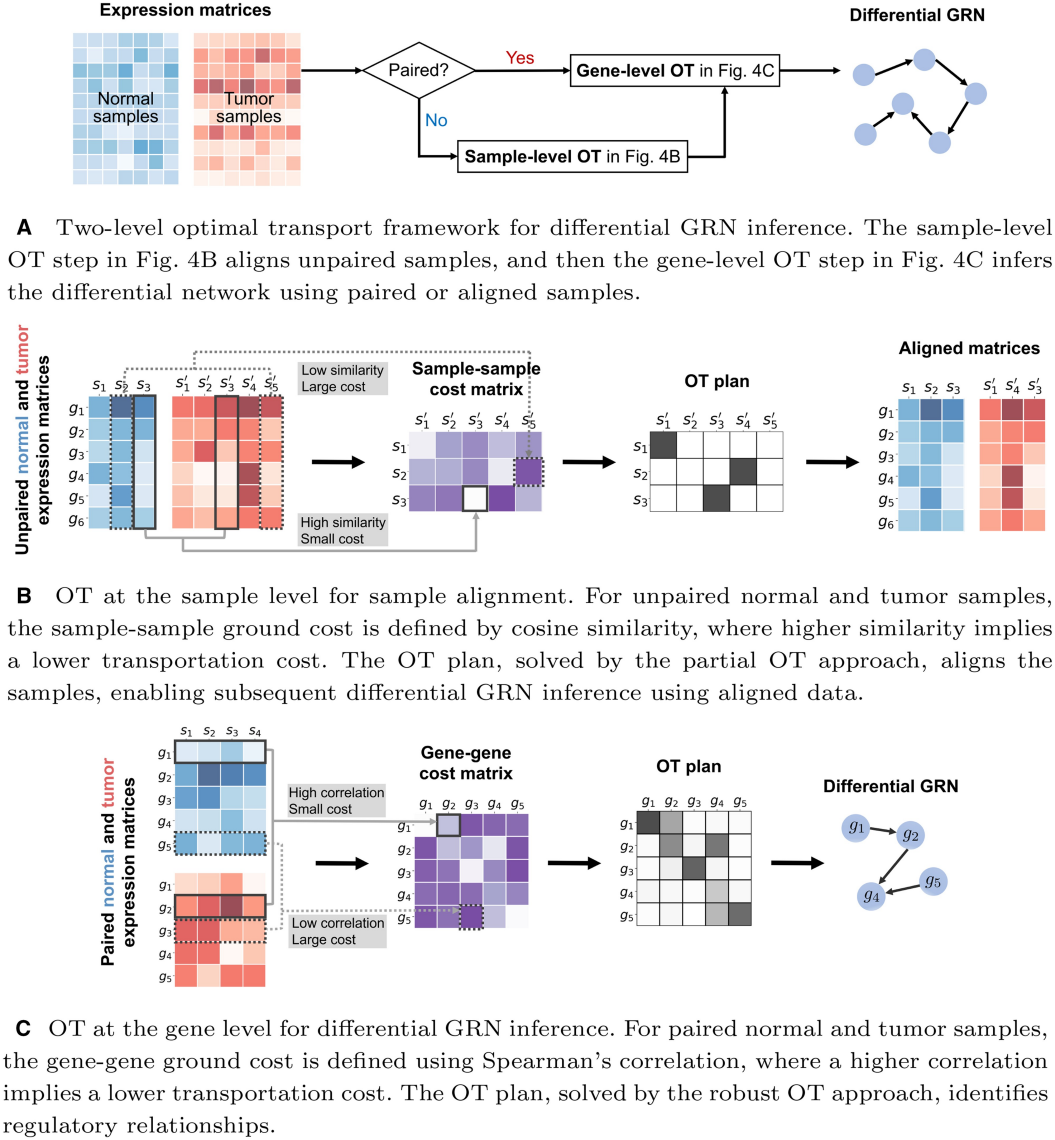
Overview of the proposed Double OT method for inferring differential GRNs, accommodating both paired and unpaired samples.

Figure 4C illustrates the gene-level OT process.

### 2.3 Two-level OT framework

The gene-level OT approach as described above requires paired samples to calculate the cost matrix between genes, and therefore is impractical when the samples are not paired. To tackle this challenge, we propose a two-level framework that first solves a pseudo-permutation matrix to match the samples and then reconstructs the differential GRN based on the aligned samples.

Our sample matching approach is motivated by the biological observation that, for an individual, only a small subset of genes typically exhibits significant expression changes between different states or conditions. Therefore, samples with similar overall expression profiles are more likely to be matched across states. This intuition can be formalized using optimal transport theory.

In this sample alignment problem, typically only a subset of the total samples need to be matched. Therefore, we employ the partial OT strategy (3) to facilitate meaningful alignments without forcing matches where none exist. Let $x_{i}$ denote the *i*th column of X, representing the *i*th sample in X, and similarly, let $y_{i}$ denote the *i*th sample in Y. We assign equal weight to each sample, setting $a=1_{n}$ and $b=1_{m}$. To ensure that more similar samples are aligned, we define the sample-sample cost matrix C using the cosine similarity in the global principal component (PC) space. Specifically, $c(x_{i},y_{j})=1-\text{cos}({\tilde{x}}_{i},{\tilde{y}}_{j})=1-{\tilde{x}}_{i}^{⊤}{\tilde{y}}_{j}/(||{\tilde{x}}_{i}|{|}_{2}||{\tilde{y}}_{j}|{|}_{2})$, where ${\tilde{x}}_{i}\in R^{r}$ (or ${\tilde{y}}_{j}\in R^{r}$) contains the first *r* PCs of $x_{i}$ (or $y_{j}$). Compared to the commonly used Euclidean distance, the cosine distance is scale-invariant and has become a popular similarity measure for gene expression data (Jaskowiak *et al.* 2014, Huizing *et al.* 2022).

The solution to the problem (3), denoted as $P^{*}$, is a 0–1 binary matrix with exactly *s* non-zero elements, with each row and column containing at most one non-zero element (Bai *et al.* 2023). This pseudo-permutation matrix establishes the alignment relationship between *s* pairs of normal and tumor samples. By applying $P^{*}$ to the unpaired samples, we can obtain the aligned pairs. In particular, let $\left\{i_{1},i_{2},\ldots ,i_{s}\right\}$ and $\left\{j_{1},j_{2},\ldots ,j_{s}\right\}$ be the sets of row and column indices where $P_{ij}^{*}=1$. Then, the aligned expression matrices can be constructed as $\hat{X}=[x_{i_{1}},\ldots ,x_{i_{s}}]$ and $\hat{Y}=[y_{j_{1}},\ldots ,y_{j_{s}}]$. This sample-level OT process is displayed in Fig. 4B.

Finally, by solving the robust OT problem (2) using aligned samples $\hat{X},\hat{Y}\in R^{p\times s}$, the differential GRN is constructed. The complete algorithm for differential GRN inference using the Double OT method is summarized in Algorithm 1 and visualized in Fig. 4A.

Algorithm 1 Double OT for Differential GRN Inference

**Require:**

 $X\in R^{p\times n},Y\in R^{p\times m}$  ▹ Expression matrices

**Ensure:**

1: **if**  X and Y are unpaired **then**

2:  $P^{*}\leftarrow$ the approximate solution to the partial OT problem (3) with (C,a,b) defined by $C_{ij}=c(x_{i},y_{j})=1-\text{cos}({\tilde{x}}_{i},{\tilde{y}}_{j})$, $a=1_{n}$, and $b=1_{m}$, where $x_{i}$ (or $y_{i}$) is the *i*th column of X (or Y), and ${\tilde{x}}_{i}$ (or ${\tilde{y}}_{i}$) contains the first *r* principle components of $x_{i}$ (or $y_{i}$).

3:  If n≤m, for each row *i* of $P^{*}$, find $j_{i}=\arg\max_{j}P_{ij}^{*}$; otherwise, for each column *j*, find $i_{j}=\arg\max_{i}P_{ij}^{*}$. Select *s* entries with top-*s* highest $P_{ij}^{*}$ among ${\left\{(i,j_{i})\right\}}_{i=1}^{n}$ or ${\left\{(i_{j},j)\right\}}_{j=1}^{m}$, denoted as ${\left\{(i_{k},j_{k})\right\}}_{k=1}^{s}$. Reorganize

       $X\leftarrow [x_{i_{1}},\ldots ,x_{i_{s}}],\;\;Y\leftarrow [y_{j_{1}},\ldots ,y_{j_{s}}].$

4: **else**

5:  s←n

6: **end if**

7: $T^{*}\leftarrow$ the approximate solution to the robust OT problem (2) with (C,a,b) defined by $C_{ij}=c(x_{\left(i\right)},y_{\left(j\right)})=1-|r(x_{\left(i\right)},y_{\left(j\right)})|$, $a=X1_{s}/s$, and $b=Y1_{s}/s$, where $x_{\left(i\right)}$ (or $y_{\left(i\right)}$) is the *i*th row of X (or Y).

8: **Return**  $T^{*}$, where $T_{ij}^{*}$ is the score of the regulatory link from gene *i* to gene *j*, for i,j∈{1,…,p}.

In Algorithm 1, we approximate both the sample-level and gene-level OT problems with entropic regularization to improve scalability. Specifically, we solve the partial OT problem in (3) through the Dykstra algorithm (Benamou *et al.* 2015), with computational complexity O(nm log(n+m)). We solve the robust OT problem in (2) using the unbalanced Sinkhorn–Knopp algorithm (Pham *et al.* 2020), whose complexity is $O(p^{2}\;\text{log}\;p)$. Consequently, the total time complexity of Algorithm 1 is $O(p^{2}\;\text{log}\;p+nm\;\text{log}(n+m))$. This nearly quadratic scaling in both *n* (or *m*) and *p* makes the proposed Double OT method scalable to large-scale GRN inference problems.

Remark 3 *A related method, CO-Optimal Transport (COOT) (* Redko *et al.* 2020, Demetci *et al.* 2022*), also integrally solves for sample-level and feature-level couplings. However, COOT and Double OT are designed for different goals. COOT aims to align data from heterogeneous domains (e.g. multi-omics data) using distance-based costs, while Double OT is specifically designed for GRN inference, using biologically motivated cost functions that better capture potential regulatory strength.*

### 2.4 Performance evaluation

#### 2.4.1 Competing methods

To ensure a comprehensive and diverse evaluation, we compare our double OT method with widely used GRN inference approaches from different categories as follows.

- Baseline method: randomly ranking the importance of edges (Random).
- Correlation-based methods: Spearman’s rank-based correlation (Spearman); part mutual information with path consistency algorithm (PCA-PMI) (Zhao *et al.* 2016).
- Model-based methods: least angle regression with stability selection (TIGRESS) (Haury *et al.* 2012); max–min hill-climbing Bayesian network structure learning algorithm (MMHC) (Tsamardinos *et al.* 2006); stochastic dynamical model based on transcriptional bursting (Harissa) (Herbach 2023); joint graphical lasso (JGL) (Danaher *et al.* 2014); latent differential graphical model (LDGM) (Tian *et al.* 2016).
- Machine learning-based methods: ensemble of trees using random forests (GENIE3) (Huynh-Thu *et al.* 2010); nonlinear ordinary differential equations with XGBoost (NonlinearODE) (Ma *et al.* 2020).
- OT-based methods: unbalanced CO-OT (UCOOT) (Tran *et al.* 2023); gene velocity estimation (OTVelo) (Zhao *et al.* 2024).

Implementation details for our method and the competitors are available in the Supplementary Section S2.1, available as supplementary data at *Bioinformatics* online.

#### 2.4.2 Evaluation metrics

Given normal and tumor expression matrices, each GRN inference method predicts a ranked list of putative regulatory links. By comparing these predictions with the ground truth network, we calculate the area under the receiver operating characteristic (ROC) curve and the precision-recall (PR) curve, denoted as AUROC and AUPR, respectively. In addition to global performance, we assess the accuracy of top predictions using early precision (EP), which is the fraction of true positives among the top-*K* edges, where *K* is the number of edges in the ground truth network. This metric emphasizes the accuracy of the most confident predictions.

### 2.5 Datasets

#### 2.5.1 Synthetic data

The normal expression matrix X is generated using a Gaussian distribution to simulate the baseline gene expression levels. Subsequently, we randomly select differentially expressed (DE) genes and their parent genes. The tumor expression matrix Y is then produced using a conditional Gaussian distribution according to specific regulatory functions. These mechanisms encompass both positive and negative regulations, as well as linear and nonlinear interactions, aiming to closely resemble real-world biological scenarios. The detailed data generation process is described in Supplementary Section S2.2, available as supplementary data at *Bioinformatics* online.

It is important to note that although our synthetic data adhere to a (conditional) Gaussian distribution, as used in existing studies (Xiao 2009, Thompson *et al.* 2015), our approach itself is distribution-free and does not depend on specific distributions.

#### 2.5.2 Gastric cancer data

To explore the molecular pathogenesis of gastric cancer (GC), we analyze the gene expression array data collected by Wang *et al.* (2014). The dataset, accessible through the European Genome-phenome Archive under accession code EGAS00001000597 (https://ega-archive.org/studies/EGAS00001000597), involves gene expression profiling in 100 patients diagnosed with GC. Within this cohort, 43 patients have both normal and tumor samples available, while the remaining individuals only have tumor samples because the corresponding normal samples are absent. The dataset comprises a total of $3.1\times 10^{4}$ genes. For interpretability, we use the NCBI Reference Sequence (RefSeq) Database (O’Leary *et al.* 2016) to filter well-characterized human genes (https://ftp.ncbi.nih.gov/refseq/H_sapiens/RefSeqGene/), resulting in 6276 genes for subsequent analysis.

## 3 Results

### 3.1 Simulation studies

We evaluate the effectiveness and scalability of the proposed method using synthetic data. Considering that competing methods except for graphical models are limited to handling paired samples, we directly apply these approaches and our Double OT (DOT-p) method to the paired generated data. To further demonstrate the alignment capability of Algorithm 1, we also shuffle the tumor samples and employ Double OT (DOT-u) and graphical models (i.e. JGL and LDGM) to reconstruct the GRN from these unpaired samples.

To compare each method in various network sizes and sample sizes, we consider various combinations of the number of genes (p∈{500,5000}) and the sample size (n∈{40,100}). Regarding additional data generation parameters, we set the proportion of differentially expressed (DE) genes to α∈{5%,10%,20%} for p=500 and α∈{1%,5%,10%} for p=5000. The expected number of regulators (including itself) for each DE gene is set to λ∈{2,5,8}. These settings ensure a realistic simulation of differential GRNs.

**Figure 5.**
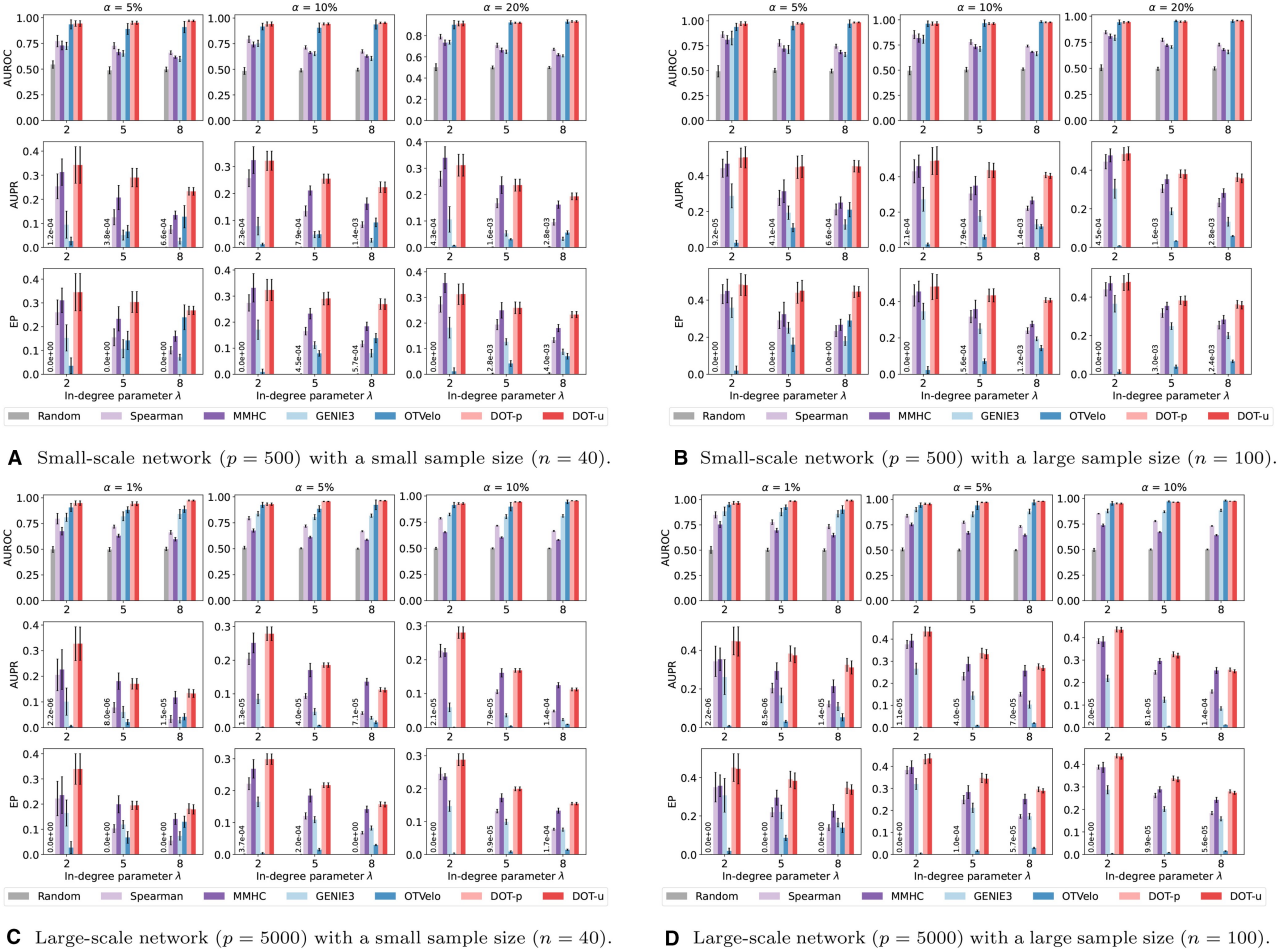
Comparison of GRN inference methods applied to unpaired (OTVelo and DOT-u) or paired (others) samples across different network sizes (panels A and B vs. C and D) and sample sizes (panels A and C vs. B and D). The performance is evaluated using three metrics, i.e. AUROC, AUPR, and EP (from top to bottom), with higher values indicating better performance. The evaluation is carried out across different proportions of DE genes α (from left to right) and in-degree parameters λ (horizontal axis). Vertical bars are the standard errors based on 10 replications. For the Random method, small values are annotated directly on the bars for clarity.

Figure 5 compares the accuracy of GRN inference methods across different scales (*p*, *n*) and parameters (α,λ). For clarity, we only present the most competitive method from each class here; the full comparison is provided in Supplementary Section S3.1, available as supplementary data at *Bioinformatics* online. Performance is comprehensively measured by AUROC, AUPR, and EP, where higher values indicate better performance. Our DOT-p/u method, as shown in Fig. 5, exhibits superior performance in most cases, particularly for large-scale networks (e.g. Fig. 5C and D). In the case of small-scale networks, the Bayesian method, MMHC, occasionally achieves slightly higher accuracy when λ=2 (see Fig. 5A). However, its advantage diminishes with increasing λ, attributed to the increasing complexity when inferring the posterior distribution with more conditioned variables. Conversely, our DOT-p/u method performs well in managing multi-wise regulation.

Compared to the Spearman method, which directly uses correlation as edge importance, our approach consistently outperforms it, especially when the sample size is relatively small. This indicates that OT effectively reduces the noise in the original correlation matrix, thereby better revealing the true signals. Additionally, the performance of machine learning-based methods like GENIE3 decreases with reduced sample sizes, further highlighting the advantages of our approach in common biological scenarios of insufficient samples. While another OT-based method, OTVelo, achieves comparable AUROC to Double OT, its performance on AUPR and EP is much lower, indicating limited ability to prioritize true regulatory interactions in imbalanced settings.

Moreover, the DOT-u applied to unpaired samples consistently achieves competitive or even identical performance compared to DOT-p applied to paired samples. This success can be attributed not only to the high accuracy rate of partial OT in sample alignment, but also to the robustness of unbalanced OT in differential GRN inference. More detailed results on sample alignment are available in Supplementary Section S3.2, available as supplementary data at *Bioinformatics* online.

**Figure 6.**
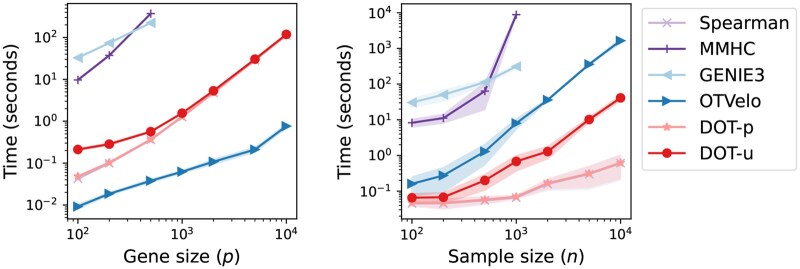
Average running time (seconds) of GRN inference methods versus increasing network sizes *p* (left panel, n=100) and sample sizes *n* (right panel, p=100) in the log-log scale. Shaded regions are the standard errors based on 10 replications.

Figure 6 presents the average computational time of each GRN inference method as the number of genes or samples increases. Detailed runtime results for various gene and sample sizes are reported in Supplementary Section S3.3, available as supplementary data at *Bioinformatics* online. Our DOT-p/u methods show great scalability in both *p* and *n*. DOT-p is only slightly slower than Spearman, and is much faster than model-based MMHC and machine learning-based GENIE3. This suggests that the main computational bottleneck of DOT-p lies in computing the ground cost matrix, which could be sped up through parallel or distributed computing. DOT-u requires more time than DOT-p due to the sample alignment step, but it still scales better than OTVelo as *n* increases. Overall, our approach achieves a decent balance between efficiency and accuracy, making it well suited for high-dimensional, large-scale datasets.

We also perform sensitivity analyses and robustness tests, with detailed results provided in Supplementary Sections S3.4 and S3.5, available as supplementary data at *Bioinformatics* online, respectively.

### 3.2 Gastric cancer data analysis

We assess the proposed method on the gastric cancer dataset from three key perspectives:

- Accuracy: if it can reconstruct a more accurate gastric cancer GRN compared to other methods.
- Coverage: if it can achieve a broader coverage of recorded regulation links than other methods.
- Exploration: if it can reveal new biological insights into the regulatory mechanisms underlying gastric cancer.

To answer the first question, we use the GC pathway (https://www.kegg.jp/pathway/hsa05226) from the Kyoto Encyclopedia of Genes and Genomes (KEGG) database (Kanehisa *et al.* 2017) as the reference for evaluation. In this small-scale reference network, 142 genes were annotated as gastric cancer genes and 431 edges were annotated as regulatory interactions. By intersecting these genes with our GC dataset, the network comprises 104 genes and 226 edges. Although this database may not be exhaustive, it serves as a valuable benchmark network containing cutting-edge knowledge. We evaluate the performance of GRN inference on this specific subset of genes. The comparison results of our double OT and other methods are reported in Tables 1 and 2. Here, DOT-u first shuffles the 100 tumor samples and applies Algorithm 1 on the unpaired and unbalanced samples. From Tables 1 and 2, we observe that DOT-p outperforms other GRN inference methods with respect to (w.r.t.) all three evaluation criteria. Moreover, the DOT-u using unpaired samples also exhibits superior accuracy. Detailed alignment results for the GC dataset are included in Supplementary Section S3.1, available as supplementary data at *Bioinformatics* online. We also evaluate DOT-u on single-cell RNA sequencing data of gastric cancer (Kang *et al.* 2022). The results show that DOT-u can achieve better performance (EP = 0.108) on higher-resolution data and remains competitive in unpaired settings without true sample correspondences. It is detailed in Supplementary Section S3.6, available as supplementary data at *Bioinformatics* online. In the following, we focus on the double OT method with paired samples (DOT-p) for more precise analyses.

**Table 1.** Comparison of methods requiring paired samples in inferring gastric cancer KEGG pathway w.r.t. AUROC, AUPR, and EP (the higher the better).^a^

Metrics	AUROC	AUPR	EP
Random	0.501	0.021	0.018
Spearman	0.513	0.021	0.009
PCA-PMI	*0.536*	*0.023*	*0.027*
TIGRESS	*0.530*	*0.024*	*0.027*
MMHC	0.510	*0.023*	*0.031*
GENIE3	0.524	0.022	0.022
NonlinearODE	0.521	0.021	0.004
DOT-p	***0.551***	***0.029***	***0.040***

a The top-3 results of each metric are in italics. The best is in bold.

**Table 2.** Comparison of methods applicable to unpaired samples in inferring gastric cancer KEGG pathway w.r.t. AUROC, AUPR, and EP (the higher the better).^a^

Metrics	AUROC	AUPR	EP
Harissa	***0.571***	0.024	0.004
JGL	0.509	*0.025*	***0.031***
LDGM	0.540	*0.025*	0.027
UCOOT	*0.546*	0.023	0.013
OTVelo	0.515	0.023	***0.031***
DOT-u	*0.556*	***0.026***	***0.031***

a The top-3 results of each metric are in italics. The best is in bold.

We then proceed to assess the coverage of recorded regulatory links within the reconstructed GRN. In this analysis, we use the TFLink gateway (Liska *et al.* 2022) comprising human transcription factor–target gene (TF–TG) interactions as a reference, to investigate how many known TF–TG links existed in the top-ranked positive edges for each method. The results are relegated to Supplementary Section S3.7, available as supplementary data at *Bioinformatics* online, illustrating the broader coverage achieved by our double OT method.

**Figure 7.**
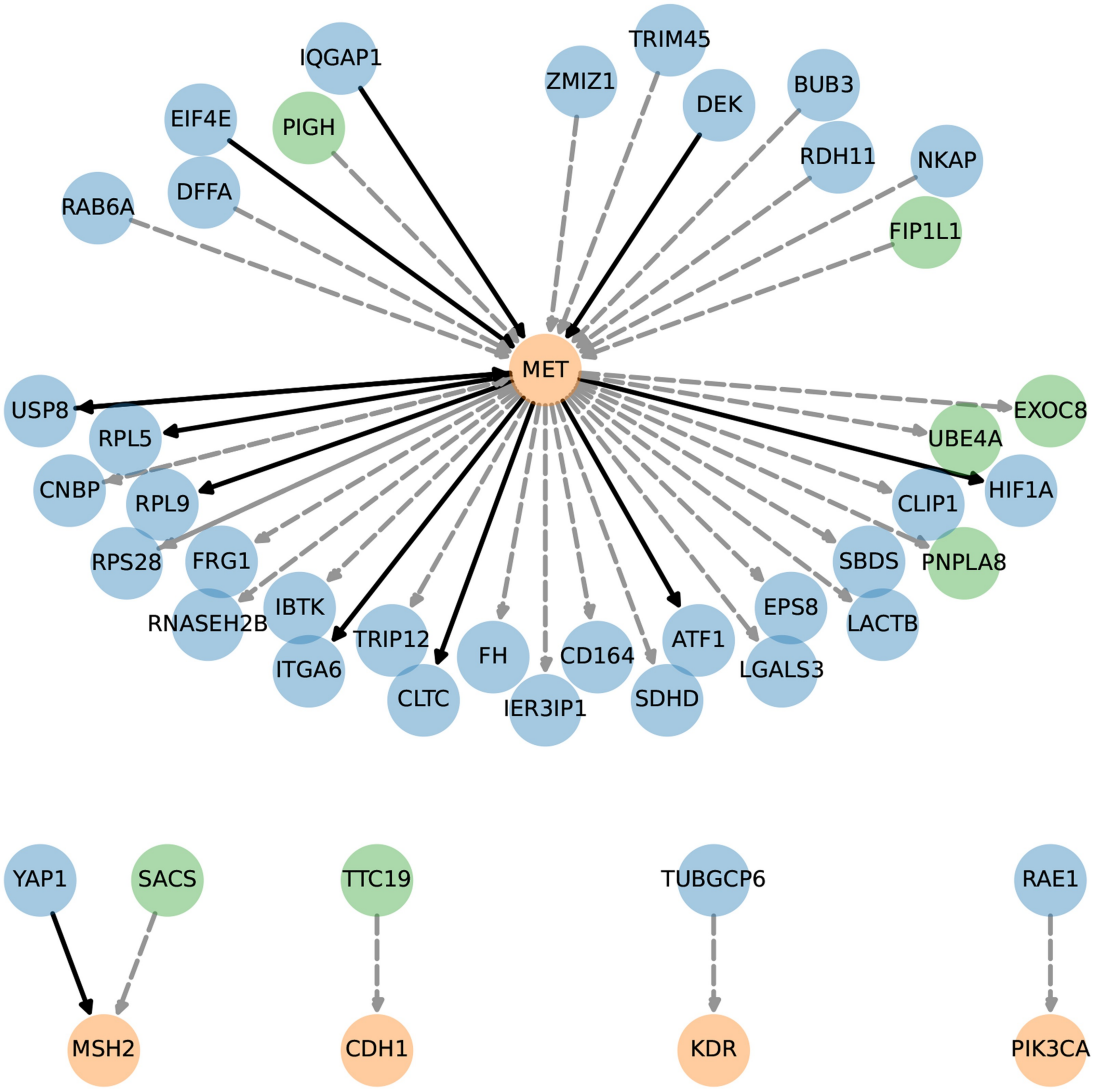
Gastric cancer biomarker-linked subnetwork constructed by DOT-p. The orange, blue, and green nodes are biomarker genes, recorded GC-related genes (e.g. oncogenes, tumor suppressor genes, and dysregulated genes), and newly discovered GC-related genes, respectively. The solid and dashed edges are recorded and newly discovered regulatory relationships, respectively.

In addition, we extract a subnetwork that includes the edges directly linked to the GC biomarker genes (Choi *et al.* 2022, Park *et al.* 2023) among the top-5000 edges constructed by our DOT-p method, as shown in Fig. 7. In this subnetwork, nearly all genes have been validated as being related to gastric cancer. Moreover, many of the regulatory relationships represented by these edges have been corroborated in the existing literature. References for the validated GC-related genes and regulatory links can be found in Supplementary Section S3.8, available as supplementary data at *Bioinformatics* online. Furthermore, proto-oncogene *Mesenchymal-Epithelial Transition (MET)*, a prototypical receptor tyrosine kinase, is recognized as the hub node within the subnetwork shown in Fig. 7. Its overexpression is mostly noted in dysplasia and precancerous intestinal metaplasia, illustrating its critical role in the early phase of the oncogenesis of GC (Sun *et al.* 2012). Targeting inhibitors against *MET* also presents promising avenues for drug development in the context of gastric cancer (El Darsa *et al.* 2020). Furthermore, we conduct gene enrichment analysis for 1033 genes linked to top-5000 edges; see Supplementary Section S3.9 for details, available as supplementary data at *Bioinformatics* online. The pathway, human papillomavirus infection, is the most significant one. The connection between human papillomavirus infection and gastric cancer, although unexpected, has been reported by prior studies (Zeng *et al.* 2016). These findings not only further support our method but also provide novel biological discoveries into the regulatory mechanisms of gastric cancer.

To better reflect real-world scenarios, where unpaired samples may not have true matching relationships, we further evaluate the proposed method using data from independent sources and refer to Supplementary Section S3.10 for details, available as supplementary data at *Bioinformatics* online.

## 4 Discussion

In this study, we conceptualize changes in gene expression between states as a mass transport problem and propose a two-level OT framework, named double OT, to infer large-scale differential GRNs for paired or unpaired samples. This method determines edge scores by solving the robust OT problem and handles unpaired samples by incorporating a partial OT-based sample alignment step. To our knowledge, this is the first approach that explicitly models gene regulation as a mass transportation problem from the perspective of OT theory.

Extensive experiments show that our approach, using either paired or unpaired samples, outperforms state-of-the-art GRN inference methods, many of which are limited to paired samples, in both effectiveness and efficiency. By applying the double OT method to a gastric cancer dataset, we also uncover novel biological insights into the regulatory mechanisms involved in gastric cancer.

Our work has limitations and can be improved or extended in several ways. First, due to the nature of optimal transport, the inferred links in our GRN are more likely to reflect associations rather than actual or direct gene regulatory interactions. Future work could refine this by incorporating additional biological constraints or causal inference techniques. Second, while this work only focuses on gene expression profiles, integrating multi-omic data could potentially yield more precise GRNs. Third, while our method directly models gene transitions between states, combining it with intra-state comparisons through the Fused Gromov–Wasserstein framework (Vayer *et al.* 2020) would offer a more comprehensive view of gene relations. Developing a computationally efficient method for this combination remains a challenge and is left for future work. Fourth, this work assumes static regulatory relationships between states (e.g. normal versus tumor), which may not fully capture the dynamic nature of gene regulation over time. Extending the double OT method to incorporate temporal data could provide deeper insights into the dynamics of gene regulatory networks. Finally, although the proposed method can handle large-scale networks with thousands of nodes, its nearly quadratic complexity poses challenges for much larger networks. Developing fast OT solvers that better fit the characteristics of GRN problems remains an exciting direction for further investigation.

## Supplementary Material

btaf352_Supplementary_Data

## Data Availability

The data used in this study are publicly available. Accession codes or links are provided in the manuscript.
