# Supplementary material for: Double optimal transport for differential gene regulatory network inference with unpaired samples
Source: Bioinformatics. 2025 Aug 4;41(8):btaf352. doi: 10.1093/bioinformatics/btaf352 (PMC12342166; doi:10.1093/bioinformatics/btaf352)
Supplement: btaf352_Supplementary_Data [file btaf352_supplementary_data.pdf]

Supplementary Material for "Double Optimal Transport for Differential Gene Regulatory Network Inference with Unpaired Samples"

Mengyu Li[1] Bencong Zhu[2*] Cheng Meng[3†] Xiaodan Fan[2‡]
[1]Institute of Statistics and Big Data,
Renmin University of China, Beijing, China
[2]Department of Statistics,
The Chinese University of Hong Kong, Hong Kong, China
[3]Center for Applied Statistics, Institute of Statistics and Big Data,
Renmin University of China, Beijing, China

The supplementary material is organized as follows. Section S1 presents a unified formulation of different optimal transport problems. In Section S2, we detail the experimental setups, including the process for generating synthetic data and the implementation of both our method and the competitors. In Section S3, we provide additional experimental results, covering sample alignment performance, the coverage of TF-TG links, and references for validated cancer-related genes and regulatory links mentioned in the manuscript.

S1 General optimal transport formulation

In Section 2.1 of our manuscript, we introduce the classical optimal transport (OT) problem and its two variants, robust OT and partial OT. These OT problems can be unified under a general formulation as follows:

$$\min_{\mathbf{T}\in\mathcal{M}_+} \underbrace{\langle \mathbf{C}, \mathbf{T}\rangle}_{\text{Transp. cost}} + \underbrace{\mathcal{D}(\mathbf{T}\mathbf{1}_{n_2}\|\boldsymbol{a}) + \mathcal{D}(\mathbf{T}^\top\mathbf{1}_{n_1}\|\boldsymbol{b})}_{\text{Marginal constraints}}, \tag{S1}$$

where $\mathcal{D}$ is a divergence function, modeling various types of distribution discrepancies. The specific choices of $\mathcal{D}$ and $\mathcal{M}_+$ define different OT problems, as detailed in Table S1.

*Joint first author
†Corresponding author, chengmeng@ruc.edu.cn
‡Corresponding author, xfan@cuhk.edu.hk

Table S1: Comparison of various optimal transport problems derived from the general formulation (S1).

	Classical OT	Robust OT	Partial OT
Marginal constraints	$\mathcal{D} = \iota_{\{=\}}$, where $\iota_{\{=\}}(\boldsymbol{p}\\|\boldsymbol{q}) = \begin{cases} 0, & \text{if } p_i = q_i, \forall i \\ +\infty, & \text{otherwise} \end{cases}$	$\mathcal{D} = \varepsilon\,\mathrm{KL}$, where $\mathrm{KL}(\boldsymbol{p}\\|\boldsymbol{q}) = \sum_i \left[p_i \log\left(\frac{p_i}{q_i}\right) - p_i + q_i\right]$	$\mathcal{D} = \iota_{\{\leq\}}$, where $\iota_{\{\leq\}}(\boldsymbol{p}\\|\boldsymbol{q}) = \begin{cases} 0, & \text{if } p_i \leq q_i, \forall i \\ +\infty, & \text{otherwise} \end{cases}$
Feasible plans	$\mathcal{M}_+ = \mathbb{R}_+^{n_1 \times n_2}$	$\mathcal{M}_+ = \mathbb{R}_+^{n_1 \times n_2}$	$\mathcal{M}_+ = \{\mathbf{T} \in \mathbb{R}_+^{n_1 \times n_2} : \sum_{i,j} T_{ij} \leq s\}$

S2 Experimental setups

S2.1 Implementation details

All experiments are implemented on a server with 256GB RAM and 64 cores Intel® Xeon® Gold 5218 CPU.

For our Double OT method, we set $s = \min(n, m)$ in the partial OT to align the smaller set of samples with a subset of the larger one. The entropic regularization parameter for partial OT is set to 0.005. We set both the marginal relaxation and entropic regularization parameters to 0.05 for solving the robust OT problem. Empirically, we find that the performance of Double OT is not sensitive to these hyperparameters. Additionally, we choose the number of principle components to $r = n + m$ to preserve much information.

For the competing methods, the Spearman method is implemented using the `SciPy` library, and MMHC is implemented from the `bnlearn` R package (Scutari, 2010). The remaining methods utilize the source codes released by their respective authors. We fine-tune the parameters of all competing methods to ensure optimized performance. For those computationally intensive methods, including model-based and machine learning-based approaches that are unable to infer networks of size 5000 in a limited time, we adopt a two-level strategy: initially applying a t-test to filter differentially expressed (DE) genes, followed by the gene regulatory network (GRN) reconstruction algorithm. For a fair comparison, self-regulation was excluded for all methods.

S2.2 Synthetic data generation

For the simulation studies in Section 3.1 of the manuscript, normal and tumor expression matrices are generated using the procedure in Algorithm 1. The signal-to-noise ratio (SNR) is set to 2 unless otherwise specified, and the remaining parameters are defined in our manuscript.

Algorithm 1 Generation of normal and tumor expression data

Require:

p ▷ Number of genes
n ▷ Number of samples
α ▷ Proportion of DE genes
λ ▷ Expected number of parents for differential genes
SNR ▷ Signal-to-noise ratio

Ensure:

```
Generate the normal matrix X = (x_ij) ∈ R^{p×n}:
for i = 1 to p do
    for j = 1 to n do
        X_ij ~ N(μ_i, σ_i) where μ_i ~ U(2,5) and σ_i ~ U(0.1,2)

Generate the tumor matrix Y = (y_ij) ∈ R^{p×n}:
Randomly select the DE index set I_diff from {1,...,p} with size ⌊αp⌋
for i = 1 to p do
    if i ∈ I_diff then
        n_{i,pa} ~ Poisson(λ − 1)                          ▷ Number of parents
        Randomly select I_{i,pa} from I_diff \ {i} with size n_{i,pa}
        I_{i,pa} = I_{i,pa} ∪ {i}                          ▷ Index set of parents
        for j = 1 to n do
            y_ij ~ N(Σ_{k∈I_{i,pa}} w_ki f_k(x_kj), σ_i/SNR), where |w_ki| ~ U(0.5,2) and the sign
    of w_ki is randomly assigned; f_k(·) is randomly selected from linear, exponential, and
    quadratic functions
    else
        for j = 1 to n do
            y_ij ~ N(x_ij, σ_i/SNR)
```

S3 Additional experimental results

S3.1 Full comparison results

Figures S1 and S2 provide the full version of Fig. 5 from the main manuscript, including all competing methods. In addition to the observations already discussed, we note that the graphical models (i.e., JGL and LDGM) perform comparably to other methods in terms of AUROC but are notably less effective in terms of AUPR and EP, indicating limitations

in detecting true positives under imbalanced conditions. One possible reason is that these models capture only indirect associations rather than direct changes in gene expression.

S3.2 Alignment performance of Double OT

To assess the efficacy of the sample alignment step in the Double OT method (DOT-u), we compare it with two competing methods: (i) Correlation-based matching (Corr), which calculates the Pearson's correlation between every pair of samples from two datasets and matches each sample with the highest correlating, unused sample from the other dataset; and (ii) Waddington-OT (WOT) (Schiebinger et al., 2019), which leverages an unbalanced variant of optimal transport for sample matching.

Synthetic data. We synthesize data using Algorithm 1 with parameters $p \in \{500, 5000\}$, $n \in \{40, 100\}$, $\alpha = 20\%$, $\lambda \in \{2, 5, 8\}$, and SNR $\in \{0.5, 1, 2, 5\}$, and shuffle the tumor samples to simulate the unpaired data. Subsequently, we apply Corr, WOT, and DOT-u methods to align the unpaired samples. Once alignment is achieved, we implement the GRN reconstruction step of Double OT to infer the network. Figure S3 presents the results of matching accuracy and network inference accuracy (i.e., AUROC, AUPR, and EP).

In Fig. S3, all methods show improved performance as the SNR increases. Notably, the DOT-u method achieves nearly perfect matching accuracy over a broad range of SNR, consistently surpassing its competitors and exhibiting superior alignment robustness under various conditions.

Gastric cancer data. The gastric cancer (GC) dataset comprises 100 tumor samples and 43 normal samples, forming 43 pairs. We focus on two alignment tasks: (i) global alignment (43 vs. 43), matching the 43 normal samples with the corresponding subset of tumor samples; and (ii) local alignment (43 vs. 100), finding the best matches for the 43 normal samples within the entire set of 100 tumor samples. Given that the GC patients are categorized into five molecular subtypes (Wang et al., 2014), we assess the performance based on both pair matching accuracy and subtype matching accuracy. The experimental results of different methods are presented in Table S2, which shows that our DOT-u method not only achieves the highest accuracy in pairing but also excels in correctly identifying molecular subtypes, demonstrating its effectiveness in both global and local alignment tasks.

S3.3 Computational time and scalability

To evaluate the scalability of the Double OT method, we measure its runtime under various combinations of gene sizes p and sample sizes n. The results are summarized in Tables S3 and S4, reporting the runtime for paired (DOT-p) and unpaired (DOT-u) sample settings,

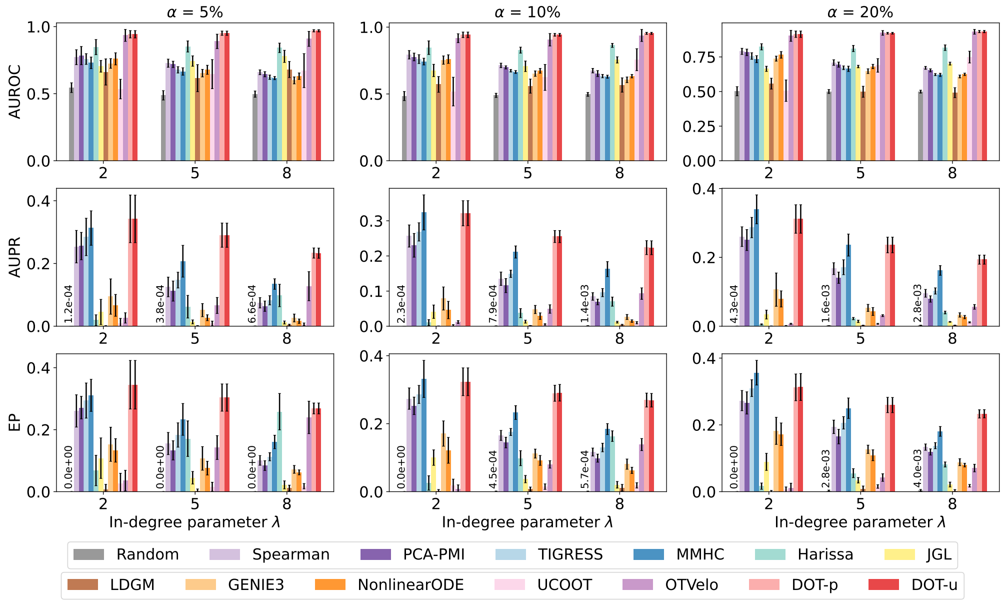

(a) Small sample size ($n = 40$).

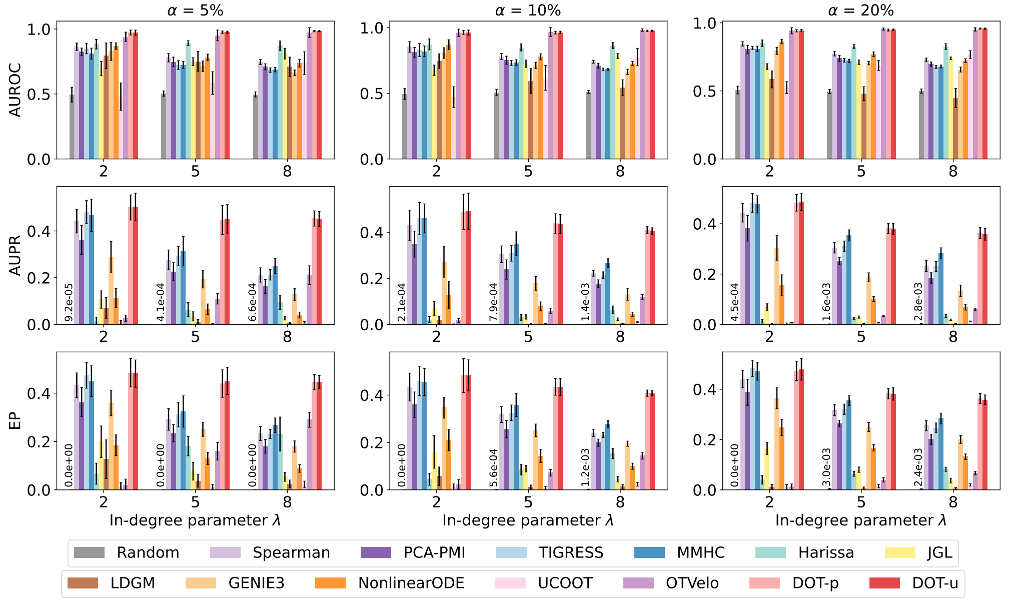

(b) Large sample size ($n = 100$).

Figure S1: Full comparison of GRN inference methods applied to unpaired (Harissa, JGL, LDGM, UCOOT, OTVelo, and DOT-u) or paired (others) samples on small-scale networks ($p = 500$). The performance is evaluated using three metrics, i.e., AUROC, AUPR, and EP (from top to bottom), with higher values indicating better performance. The evaluation is carried out across different proportions of DE genes α (from left to right) and in-degree parameters λ (horizontal axis). Vertical bars are the standard errors based on ten replications. For the Random method, small values are annotated directly on the bars for clarity.

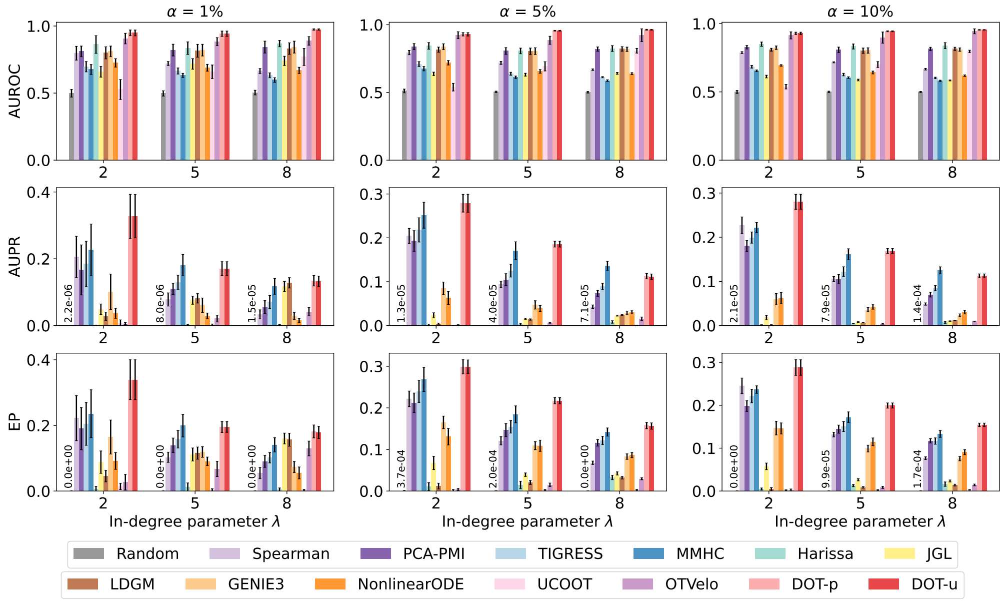

(a) Small sample size ($n = 40$).

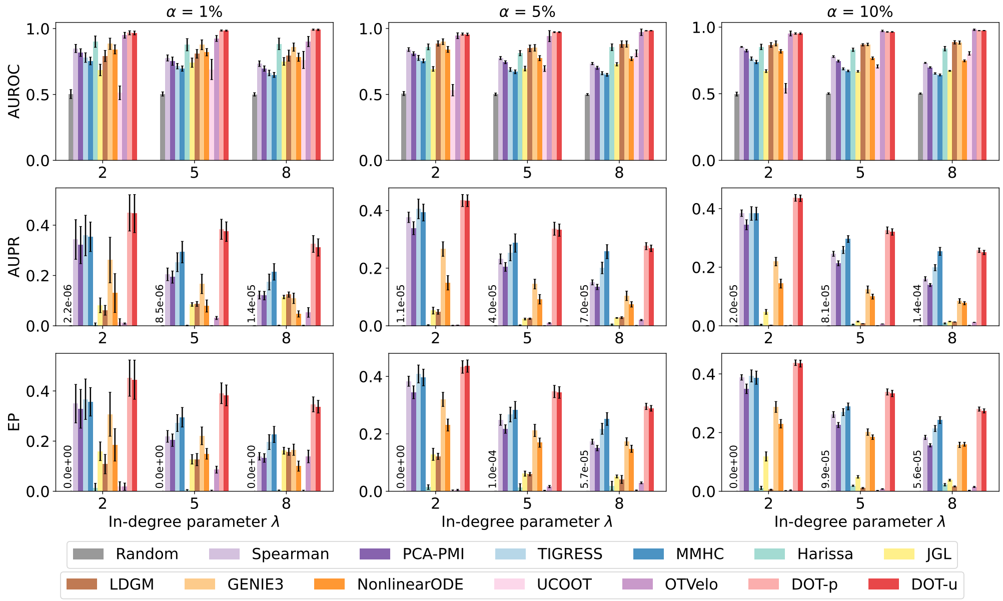

(b) Large sample size ($n = 100$).

Figure S2: Full comparison of GRN inference methods on large-scale networks ($p = 5000$). Metric definitions and evaluation settings are the same as in Fig. S1.

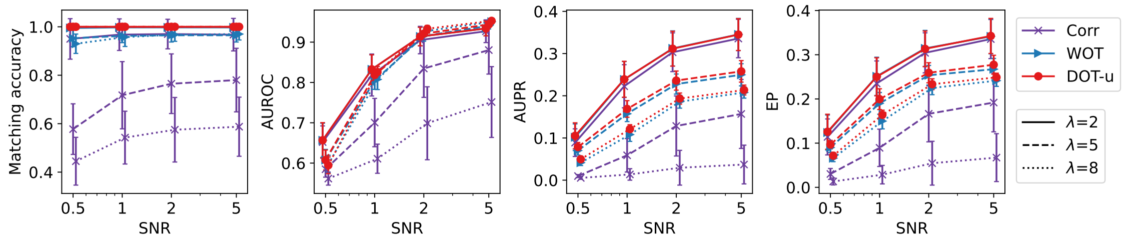

(a) Small-scale network ($p = 500$) with a small sample size ($n = 40$).

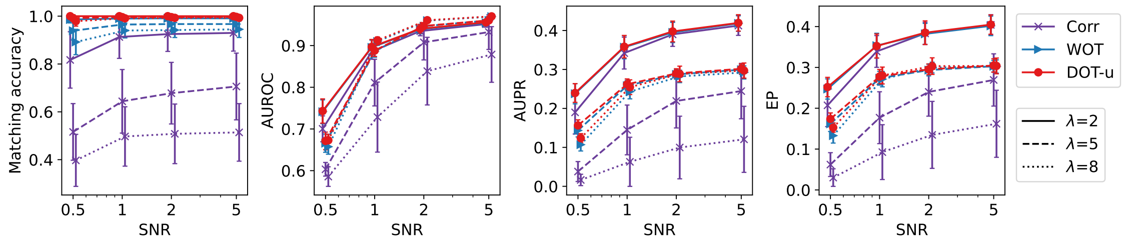

(b) Small-scale network ($p = 500$) with a large sample size ($n = 100$).

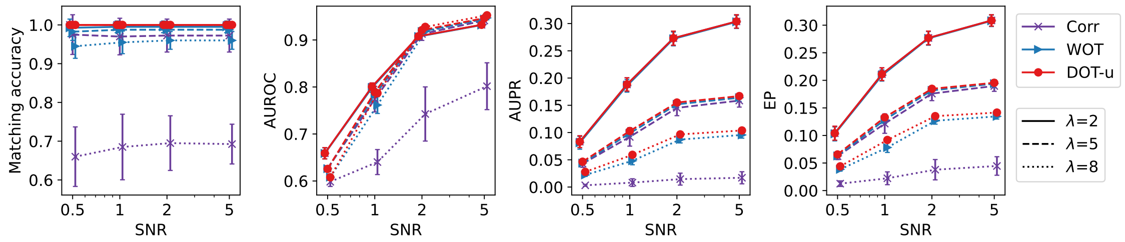

(c) Large-scale network ($p = 5000$) with a small sample size ($n = 40$).

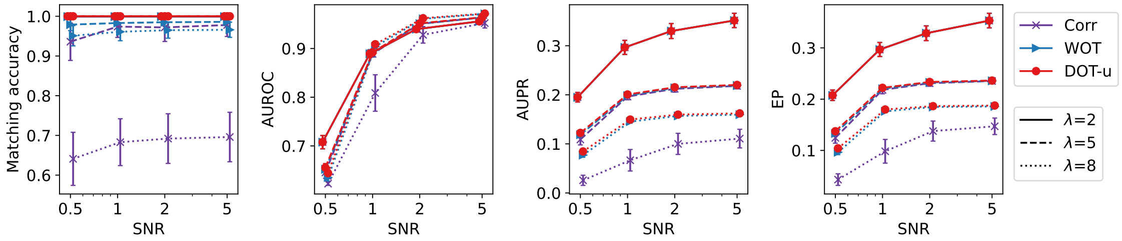

(d) Large-scale network ($p = 5000$) with a large sample size ($n = 100$).

Figure S3: Comparison of sample alignment methods across different network and sample sizes. Performance is evaluated by matching accuracy and downstream GRN reconstruction accuracy (i.e., AUROC, AUPR, and EP) versus increasing SNR. Line styles denote different methods and in-degree parameters λ. Vertical bars are the standard errors based on ten replications.

respectively. For DOT-p, the runtime is predominantly dependent on p and is less affected by n. It handles up to 10^4 genes and 10^4 samples within 2 minutes. In contrast, the runtime

Table S2: Comparison of sample alignment methods on gastric cancer data.

Method	43 vs. 43		43 vs. 100	
	Pair acc.	Subtype acc.	Pair acc.	Subtype acc.
Corr	32.6%	46.5%	32.6%	48.8%
WOT	72.1%	74.4%	53.5%	62.8%
DOT-u	86.0%	90.7%	55.8%	62.8%

of DOT-u depends on both p and n, and it can deal with 10^4 genes and thousands of samples in about 5 minutes. These results show that the Double OT method is significantly more scalable than existing methods, which generally can only manage hundreds of genes within a reasonable runtime.

Table S3: Average running time (seconds) of the Double OT method using paired samples (DOT-p) for varying gene sizes p and sample sizes n, averaged on ten replications.

$p\backslash n$	10^2	2×10^2	5×10^2	10^3	2×10^3	5×10^3
10^2	0.03	0.03	0.04	0.05	0.05	0.09
2×10^2	0.08	0.08	0.09	0.09	0.12	0.21
5×10^2	0.33	0.33	0.35	0.38	0.45	0.72
10^3	1.11	1.12	1.14	1.24	1.37	1.81
2×10^3	4.23	4.33	4.33	4.50	4.81	5.62
5×10^3	25.33	25.83	25.68	26.19	27.13	28.71
10^4	100.63	101.51	101.32	101.98	104.47	106.02

S3.4 Robustness test

To further validate the robustness of our Double OT method, we conduct experiments with data containing various levels of noise and outliers. Specifically, we consider combinations of different noise levels (i.e., SNR $\in \{4, 2, 1\}$) and outlier proportions (i.e., $\gamma \in \{5\%, 10\%, 15\%\}$). For each setting, we first generate expression matrices $\mathbf{X}$ and $\mathbf{Y}$ following Algorithm 1, and then randomly replaced $(\gamma \times 100)\%$ of elements in $\mathbf{Y}$ with one of three types of outliers:

(a) Heavy-tailed: $y_{ij} \sim t_2(x_{ij}, \sigma_i/\mathrm{SNR})$, where t_2 is a t-distribution with 2 degrees of freedom;

(b) Extreme values: $y_{ij} \leftarrow y_{ij} + c\sigma_i/\mathrm{SNR}$, where c is randomly assigned with ± 3;

(c) Bernoulli distribution: y_{ij} is a Bernoulli 0-1 random variable.

Table S4: Average running time (seconds) of the Double OT method using unpaired samples (DOT-u) for varying gene sizes p and sample sizes n, averaged on ten replications.

$p\backslash n$	10^2	2×10^2	5×10^2	10^3	2×10^3	5×10^3
10^2	0.06	0.06	0.09	0.32	1.30	5.20
2×10^2	0.09	0.10	0.16	0.42	1.69	6.91
5×10^2	0.34	0.35	0.50	0.99	3.27	11.91
10^3	1.12	1.15	1.36	2.41	6.08	23.31
2×10^3	4.25	4.38	4.75	6.39	13.83	48.72
5×10^3	25.37	25.96	26.51	29.98	46.34	230.37
10^4	100.70	101.77	103.05	109.71	137.12	343.00

The results for gene size $p = 500$, sample size $n \in \{40, 100\}$, differentially expressed gene proportion $\alpha = 10\%$, and in-degree parameter $\lambda = 5$ are shown in Fig. S4, where we observe that our method consistently outperforms other approaches and shows only a slight decline in performance as noise and outlier levels increase, demonstrating its robustness in handling data with significant noise and outliers.

S3.5 Sensitivity analysis

To assess the sensitivity of the proposed method to the marginal relaxation parameter ε, we vary ε from 10^{-3} to 1, and the performance of our Double OT method across these values is shown in Fig. S5. The results indicate that our method achieves near-optimal performance when ε is in the range of 0.02 to 0.2 and does not show significant degradation even with larger variations in this parameter. This suggests that our method is relatively robust to changes in ε.

Therefore, in the manuscript, we fixed ε at 0.05 for simplicity, without parameter tuning. However, refining the selection of ε could be a direction for future work. As shown in Fig. S5, better ε values are often associated with lower standard deviations in performance metrics. Thus, it is potential to choose ε based on network stability. Several network stability criteria have been discussed in the literature (Zhang et al., 2019, 2020).

S3.6 Validation on gastric cancer scRNA-seq data

To validate the discoveries in gastric cancer beyond bulk RNA data, we applied the DOT-u method on single-cell RNA sequencing (scRNA-seq) data from normal and tumor tissues,

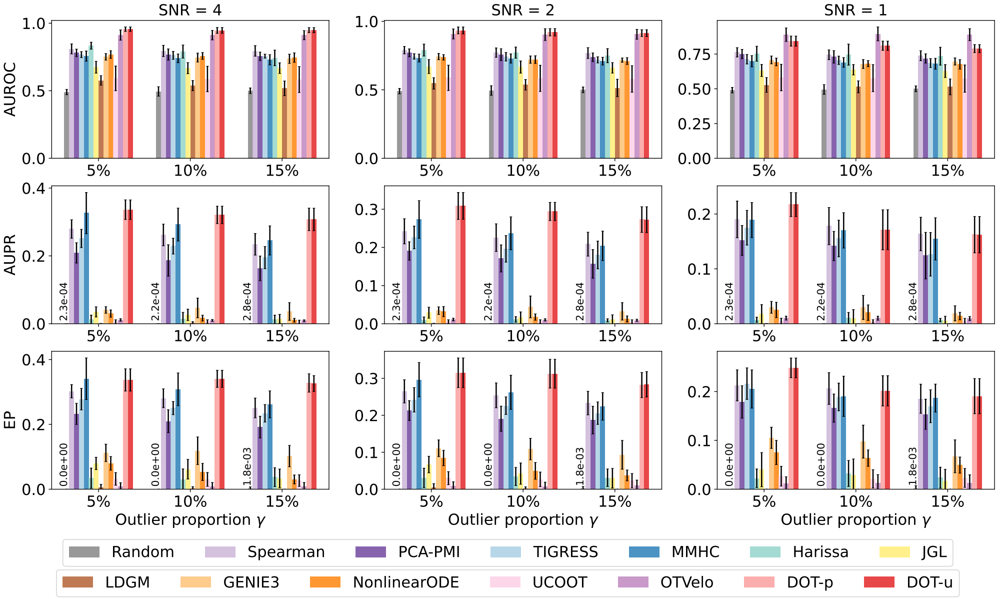

(a) $p = 500, n = 40$.

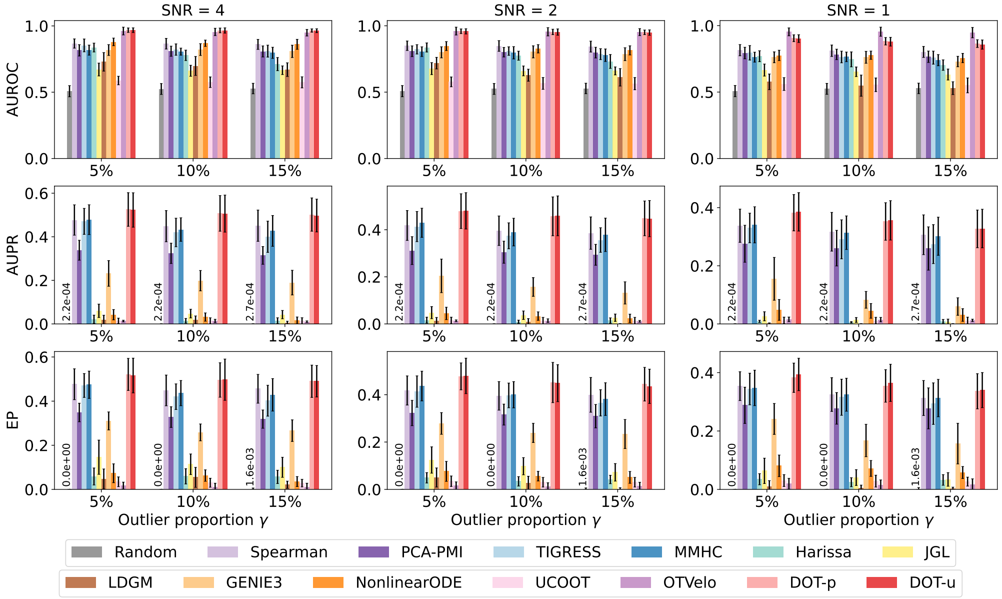

(b) $p = 500, n = 100$.

Figure S4: Comparison of GRN inference methods under various levels of noise and outliers. Each subfigure corresponds a combination of n and p, with rows indicating different performance metrics and columns indicating increasing noise levels. Horizontal axes represent increasing outlier proportions. Vertical bars show standard errors based on ten replications. For the Random method, small values are annotated directly on the bars for clarity.

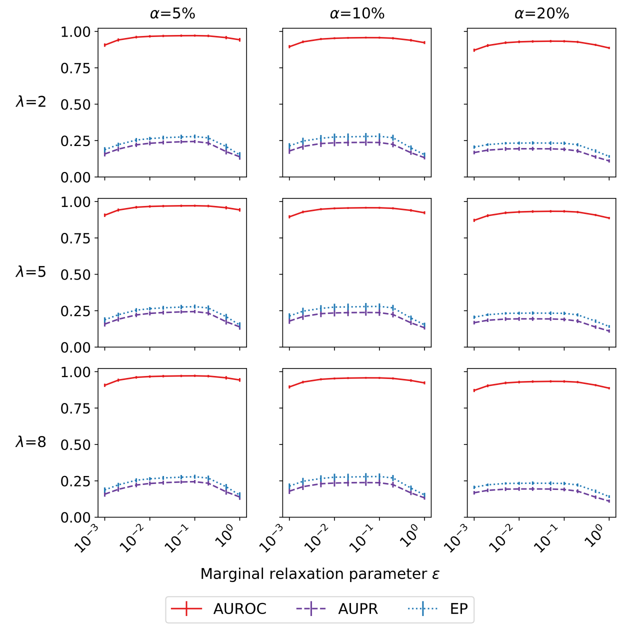

(a) $p = 500, n = 40$.

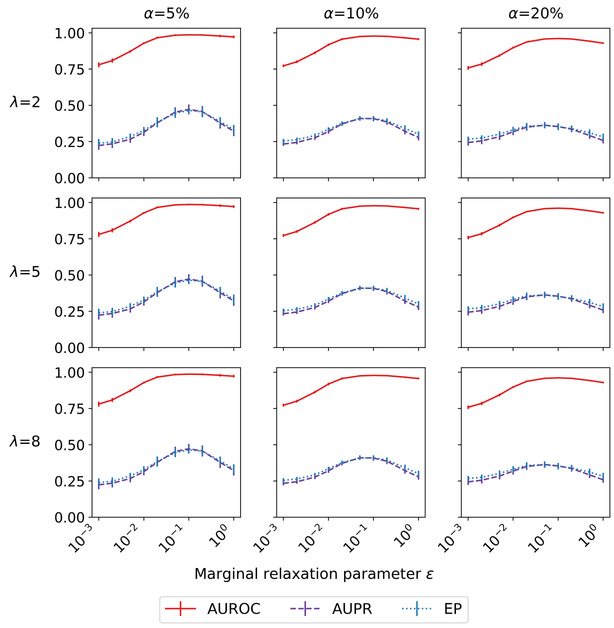

(b) $p = 500, n = 100$.

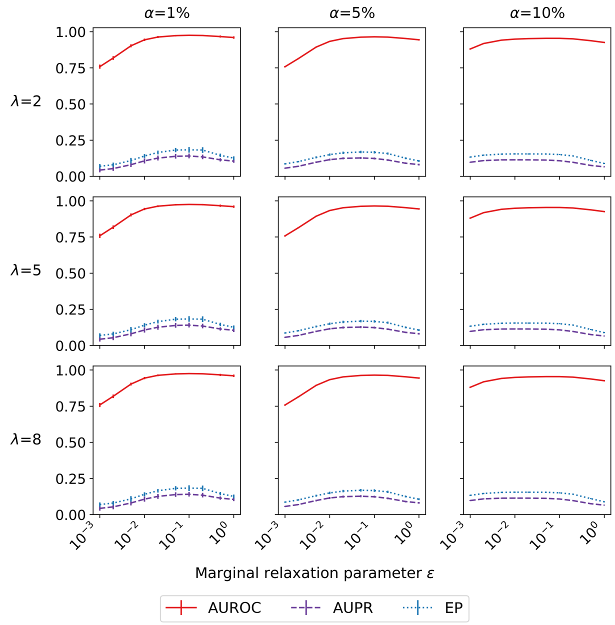

(c) $p = 5000, n = 40$.

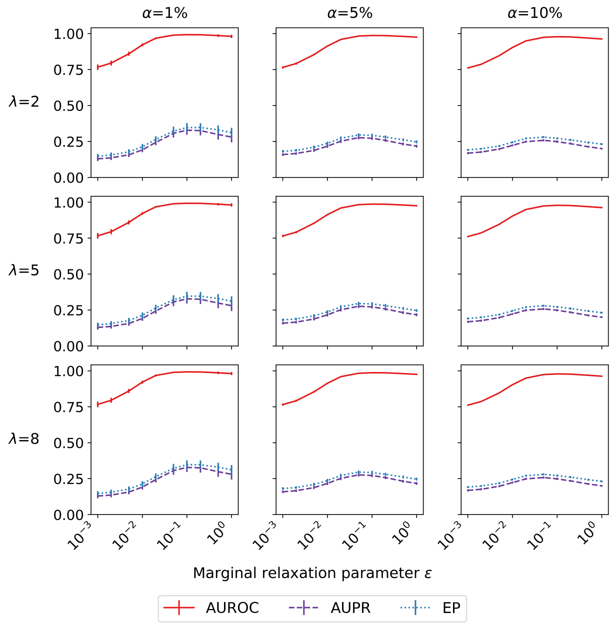

(d) $p = 5000, n = 100$.

Figure S5: Sensitivity analysis of the Double OT method to the marginal relaxation parameter ε on a log-scaled horizontal axis. Each line style represents a performance metric, and each subfigure corresponds a combination of n and p, with rows indicating specific in-degree parameters λ and columns indicating DE gene ratios α. Vertical bars show standard errors based on ten replications.

Table S5: Comparison of methods applicable to unpaired samples in inferring gastric cancer KEGG pathway using single-cell RNA sequencing data.

Metrics	AUROC	AUPR	EP
Harissa	0.476	0.022	*0.042*
JGL	0.499	0.011	0.036
LDGM	*0.506*	0.023	0.030
UCOOT	*0.547*	*0.026*	0.018
OTVelo	0.486	*0.027*	*0.054*
DOT-u	***0.572***	***0.042***	***0.108***

* The top-3 results of each metric are in italics. The best is in bold.

downloaded from the GEO repository under accession GSE206785[1] (Kang et al., 2022). This dataset contains tens of thousands of cells from both tumor and normal tissues of 24 gastric cancer patients. After log-normalization with `Seurat` package, the genes utilized in the previous bulk RNA data are kept as input for GRN inference, and the same KEGG gastric cancer pathway is used as the reference network.

For comparison, we implemented our DOT-u method and competing methods capable of handling unpaired single cells. Table S5 presents the results validated by the known gastric cancer pathway. We observe that DOT-u remains highly competitive in unpaired settings without true sample correspondences, and even outperforms several methods specifically designed for single-cell data, such as Harissa and OTVelo. We also note that DOT-u achieves better performance on scRNA-seq data compared to bulk RNA array data. This can be attributed to the fact that scRNA-seq data from different tissues of the same patient mitigates the patient-specific effects commonly observed in bulk tissue profiling.

S3.7 Coverage of TF-TG links

To evaluate how well the reconstructed gastric cancer GRN captures known regulatory interactions, we use human transcription factor-target gene (TF-TG) links in the TFLink gateway[2] (Liska et al., 2022) as references. Specifically, we count the number of TF-TG links present among the top-K positive edges for each method. Due to the inability of other methods to construct networks exceeding 6×10^3 nodes within a limited time, we only compare our Double OT (DOT-p) method with the Random baseline and the Spearman method, on paired

[1] http://ncbi.nlm.nih.gov/geo/query/acc.cgi?acc=GSE206785

[2] https://tflink.net/

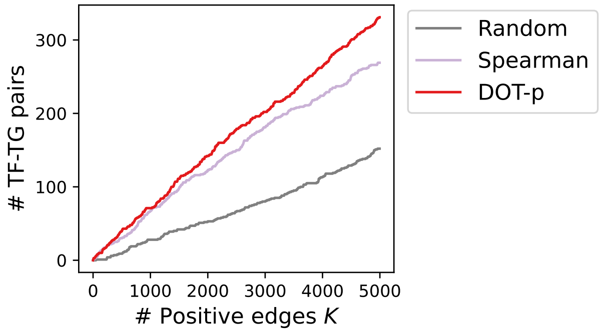

Figure S6: The number of TF-TG links covered by different methods in the top-K positive edges.

samples. Figure S6 shows the number of TF-TG links identified as K increases, highlighting the broader coverage of our DOT-p method, further validating its effectiveness.

S3.8 References for existing regulatory relationships

Tables S6 and S7 provide references for GC-related genes and regulatory links illustrated in Fig. 7 of the manuscript.

S3.9 KEGG pathway enrichment analysis

We investigate whether the genes connected to the top-5000 identified edges represent meaningful functional annotation in a biological sense by employing the Database for Annotation, Visualization, and Integrated Discovery (DAVID) (Dennis et al., 2003). Annotation terms at a 0.05 threshold applied to adjusted p-values are selected (Benjamini and Hochberg, 1995). Table S9 provides the complete list of significant KEGG pathways, where Human papillomavirus infection is the most significant. The connection between Human papillomavirus infection and gastric cancer, although unexpected, has been reported by prior studies (Zeng et al., 2016; Sofiani et al., 2023). Among the top-20 pathways, a majority are associated with signaling and various types of cancers, illustrating that the edges we have identified are related to gene regulation among cancer cells.

S3.10 Evaluation on data from independent sources

To evaluate the applicability of our method on unpaired samples without existing true matching relationships, we test the proposed approach using tumor and normal data from independent sources. Specifically, we collect gene expression data of normal tissue from 25 additional

Table S6: References for validated GC-related genes in the GC biomarker-linked subnetwork constructed by Double OT.

Gene	PubMed ID	Gene	PubMed ID
NKAP	31802890	TRIP12	29849123
RDH11	18070016	CLTC	35686089
BUB3	12692836	FH	31138787
DEK	31766266	IER3IP1	34715892
TRIM45	34168979	CD164	38201623
ZMIZ1	38332346	SDHD	12883710
CNBP	31718709	ATF1	35397606
USP8	32848421	LGALS3	36430322
IQGAP1	11289714	EPS8	32147678
EIF4E	23588929	LACTB	36078157
DFFA	29091952	SBDS	31218757
RAB6A	18070016	CLIP1	36147922
MET	37510761	HIF1A	25686741
RPL5	32194724	YAP1	27835600
RPL9	20331625	TUBGCP6	37438735
RPS28	33391486	RAE1	38417691
FRG1	28947680	MSH2	37510761
RNASEH2B	24812152	CDH1	35327345
IBTK	24489837	KDR	35327345
ITGA6	33335550	PIK3CA	36750994

gastric cancer patients, sourced from the GSE13195 dataset[3] in the Gene Expression Omnibus (GEO). We combine these 25 independent normal samples with 100 tumor samples from our original gastric cancer dataset.

For comparison, we compare our DOT-u method with competing methods capable of unpaired samples. Table S8 presents the results validated by the known gastric cancer pathway. We observe that DOT-u still outperforms others and achieves results comparable to the original counterparts in Table 2 of the manuscript.

[3] https://www.ncbi.nlm.nih.gov/geo/query/acc.cgi?acc=gse13195

Table S7: References for validated regulatory relationships in the GC biomarker-linked subnetwork constructed by Double OT.

Regulatory gene	Target gene	PubMed ID (or DOI)
DEK	MET	36124642
USP8	MET	25744385
IQGAP1	MET	36766826
EIF4E	MET	22236867
MET	USP8	25744385
MET	RPL5	10.1002/jex2.39
MET	RPL9	10.1002/jex2.39
MET	ITGA6	10.12892/ejgo4468.2019
MET	CLTC	35859795
MET	ATF1	17724745
MET	HIF1A	35859795
YAP1	MSH2	36124642

Table S8: Comparison of different methods on independent samples in inferring gastric cancer KEGG pathway w.r.t. AUROC, AUPR, and EP (the higher the better).

Metrics	AUROC	AUPR	EP
Harissa	0.529	0.025	0.039
JGL	0.506	0.027	0.026
LDGM	0.512	0.024	0.013
UCOOT	0.498	0.024	0.026
OTVelo	0.442	0.021	0.013
DOT-u	**0.535**	**0.029**	**0.065**

* The best result is in bold.

Table S9: Full KEGG pathway enrichment results for the genes connected to top-5000 edges identified by the Double OT method.

**KEGG term**	**Adjusted p-value**
hsa05165:Human papillomavirus infection	3.98E-05
hsa05200:Pathways in cancer	4.82E-05
hsa05211:Renal cell carcinoma	5.68E-05
hsa05205:Proteoglycans in cancer	0.000427
hsa05208:Chemical carcinogenesis - reactive oxygen species	0.000446
hsa04510:Focal adhesion	0.001835
hsa04660:T cell receptor signaling pathway	0.002596
hsa05161:Hepatitis B	0.008834
hsa04151:PI3K-Akt signaling pathway	0.011896
hsa05169:Epstein-Barr virus infection	0.014222
hsa04068:FoxO signaling pathway	0.014222
hsa04012:ErbB signaling pathway	0.014222
hsa05203:Viral carcinogenesis	0.014222
hsa04512:ECM-receptor interaction	0.017564
hsa01521:EGFR tyrosine kinase inhibitor resistance	0.017564
hsa05166:Human T-cell leukemia virus 1 infection	0.019759
hsa05415:Diabetic cardiomyopathy	0.023138
hsa04014:Ras signaling pathway	0.023138
hsa05010:Alzheimer disease	0.025343
hsa05213:Endometrial cancer	0.025343
hsa05220:Chronic myeloid leukemia	0.025343
hsa04210:Apoptosis	0.025343
hsa04218:Cellular senescence	0.025343
hsa05014:Amyotrophic lateral sclerosis	0.025343
hsa04714:Thermogenesis	0.025343
hsa05160:Hepatitis C	0.025343
hsa05215:Prostate cancer	0.025343
hsa05022:Pathways of neurodegeneration - multiple diseases	0.025343
hsa05225:Hepatocellular carcinoma	0.025343
hsa04066:HIF-1 signaling pathway	0.02597
hsa05226:Gastric cancer	0.02597
hsa04211:Longevity regulating pathway	0.026575

KEGG term	Adjusted p-value
hsa05167:Kaposi sarcoma-associated herpesvirus infection	0.030234
hsa04071:Sphingolipid signaling pathway	0.030269
hsa05218:Melanoma	0.030269
hsa05222:Small cell lung cancer	0.032212
hsa04611:Platelet activation	0.036147
hsa04932:Non-alcoholic fatty liver disease	0.036147
hsa04625:C-type lectin receptor signaling pathway	0.036147
hsa04910:Insulin signaling pathway	0.041151
hsa05202:Transcriptional misregulation in cancer	0.041287
hsa05221:Acute myeloid leukemia	0.041877
hsa05132:Salmonella infection	0.041877
hsa05100:Bacterial invasion of epithelial cells	0.041921
hsa05016:Huntington disease	0.042735
hsa05418:Fluid shear stress and atherosclerosis	0.044854
hsa04142:Lysosome	0.049964

References

Benjamini, Y. and Hochberg, Y. (1995). Controlling the false discovery rate: a practical and powerful approach to multiple testing. *J. R. Stat. Soc. Ser. B Stat. Methodol.*, 57(1):289–300.

Dennis, G. et al. (2003). DAVID: database for annotation, visualization, and integrated discovery. *Genome Biol.*, 4(9):1–11.

Kang, B., Camps, J., Fan, B., Jiang, H., Ibrahim, M. M., Hu, X., Qin, S., Kirchhoff, D., Chiang, D. Y., Wang, S., et al. (2022). Parallel single-cell and bulk transcriptome analyses reveal key features of the gastric tumor microenvironment. *Genome Biol.*, 23(1):265.

Liska, O. et al. (2022). TFLink: an integrated gateway to access transcription factor–target gene interactions for multiple species. *Database*, 2022:baac083.

Schiebinger, G. et al. (2019). Optimal-transport analysis of single-cell gene expression identifies developmental trajectories in reprogramming. *Cell*, 176(4):928–943.

Scutari, M. (2010). Learning Bayesian networks with the bnlearn R package. *J. Stat. Softw.*, 35:1–22.

Sofiani, V. H. et al. (2023). The complexity of human papilloma virus in cancers: A narrative review. *Infect. Agents Cancer*, 18(1):13.

Wang, K. et al. (2014). Whole-genome sequencing and comprehensive molecular profiling identify new driver mutations in gastric cancer. *Nat. Genet.*, 46(6):573–582.

Zeng, Z.-m. et al. (2016). Human papillomavirus as a potential risk factor for gastric cancer: a meta-analysis of 1,917 cases. *Onco Targets Ther.*, pages 7105–7114.

Zhang, J., Liu, J., Lee, D., Lou, S., Chen, Z., Gürsoy, G., and Gerstein, M. (2020). DiNeR: a differential graphical model for analysis of co-regulation network rewiring. *BMC Bioinformatics*, 21(281):1–15.

Zhang, X.-F., Ou-Yang, L., Yan, T., Hu, X. T., and Yan, H. (2019). A joint graphical model for inferring gene networks across multiple subpopulations and data types. *IEEE Trans. Cybern.*, 51(2):1043–1055.
